# Learning to play golf for elderly people with subjective memory complaints: feasibility of a single‐blinded randomized pilot trial

**DOI:** 10.1186/s12883-021-02186-9

**Published:** 2021-05-17

**Authors:** Julia K. Stroehlein, Solveig Vieluf, Philipp Zimmer, Alexander Schenk, Max Oberste, Christian Goelz, Franziska van den Bongard, Claus Reinsberger

**Affiliations:** 1 Department of Sports and Health, Institute of Sports Medicine, Paderborn University, Warburger Straße 100, 33098 Paderborn, Germany; 2 Department for Performance and Health, Institute for Sport and Sport Science, Technical University Dortmund, Otto-Hahn-Straße 3, 44227 Dortmund, Germany; 3 Institute of Medical Statistics and Computational Biology, Medical Faculty and University Hospital of Cologne, University of Cologne, Robert-Koch-Straße 10, 50931 Cologne, Germany

**Keywords:** Golf, Subjective memory complaints, Alzheimer’s Disease, Cognitive Performance, Kynurenine pathway

## Abstract

**Background:**

Subjective Memory Complaints (SMC) in elderly people due to preclinical Alzheimer’s Disease may be associated with dysregulation of the Kynurenine Pathway (KP), with an increase in neurotoxic metabolites that affect cognition. Golf is a challenging sport with high demands on motor, sensory, and cognitive abilities, which might bear the potential to attenuate the pathological changes of preclinical AD. This trial investigated the feasibility of learning to play golf for elderly with cognitive problems and its effects on cognitive functions and the KP.

**Methods:**

In a 22-week single-blinded randomized controlled trial, elderly people with SMC were allocated to the golf (*n* = 25, 180 min training/week) or control group (*n* = 21). Primary outcomes were feasibility (golf exam, adherence, adverse events) and general cognitive function (Alzheimer’s Disease Assessment Scale). Secondary outcomes include specific cognitive functions (Response Inhibition, Corsi Block Tapping Test, Trail Making Test), KP metabolites and physical performance (6-Minute-Walk-Test). Baseline-adjusted Analysis-of-Covariance was conducted for each outcome.

**Results:**

42 participants were analyzed. All participants that underwent the golf exam after the intervention passed it (20/23). Attendance rate of the golf intervention was 75 %. No adverse events or drop-outs related to the intervention occurred. A significant time*group interaction (*p* = 0.012, F = 7.050, Cohen’s d = 0.89) was found for correct responses on the Response Inhibition task, but not for ADAS-Cog. Moreover, a significant time*group interaction for Quinolinic acid to Tryptophan ratios (*p* = 0.022, F = 5.769, Cohen’s d = 0.84) in favor of the golf group was observed. An uncorrected negative correlation between attendance rate and delta Quinolinic acid to Kynurenic acid ratios in the golf group (*p* = 0.039, *r*=-0.443) was found as well.

**Conclusions:**

The findings indicate that learning golf is feasible and safe for elderly people with cognitive problems. Preliminary results suggest positive effects on attention and the KP. To explore the whole potential of golfing and its effect on cognitive decline, a larger cohort should be studied over a longer period with higher cardiovascular demands.

**Trial registration:**

The trial was retrospectively registered (2nd July 2018) at the German Clinical Trials Register (DRKS00014921).

## Introduction

Subjective Memory Complaints (SMC) are common in the elderly population and describe self-reported difficulties with cognitive functions, while objective performance remains normal [1, 2]. The heterogeneous etiology of SMC impedes a consistent characterization [3], but two distinct groups were broadly described: One, in which SMC are caused by other factors, including psychiatric diseases [4, 5], psychological distress [6] and chronic diseases [7] and the other, in which SMC represent preclinical Alzheimer’s Disease (AD) [2, 8, 9]. In the latter group, SMC were associated with neuropathological changes, including amyloid-β deposition [10, 11] and diminished grey matter volumes in brain regions affected by AD [12]. A meta-analysis also reported an increased risk of developing dementia in elderly people experiencing SMC compared to those without symptoms [2]. Accordingly, SMC might be a risk factor for subsequent development of AD, thus making it a very interesting group to study with respect to prevention of AD.

There is some evidence that the Tryptophan (TRP) consuming Kynurenine Pathway (KP) is dysregulated in AD and its preclinical manifestation mild cognitive impairment [13, 14].

**Fig. 1:**
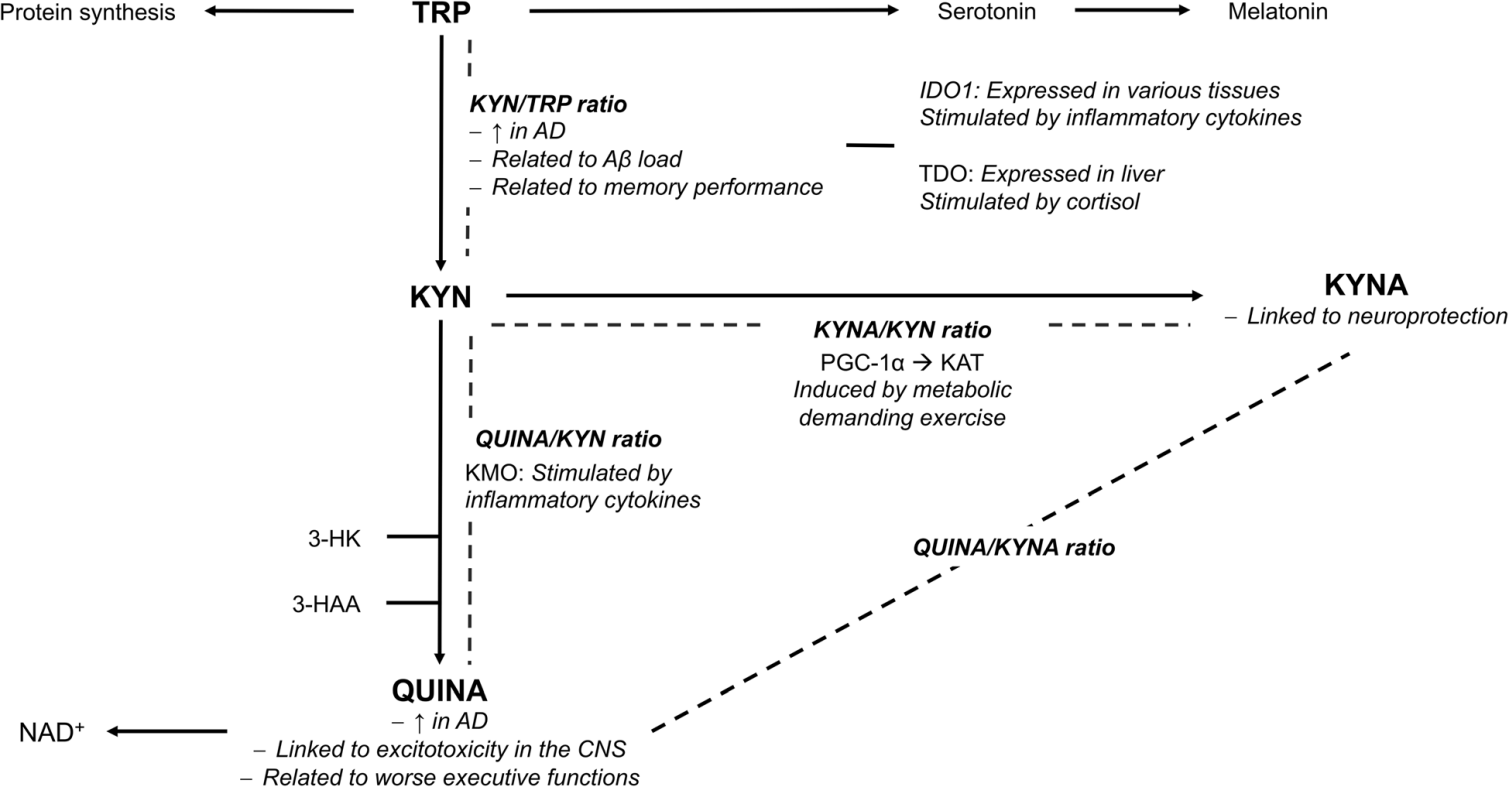
Simplified visualization of the Kynurenine Pathway. TRP: Tryptophan, KYN: Kynurenine, TDO: Tryptophan 2,3-Dioxygenase, IDO: Indoleamine-2,3-Dioxygenase 1, AD: Alzheimer’s Disease, PGC-1α: Peroxisome proliferator-activated receptor gamma coactivator 1-alpha, KAT: Kynurenine amino transferase, KYNA: Kynurenic acid, QUINA: Quinolinic acid, KMO: Kynurenine 3-monooxygenase, 3-HK: 3-hydroxykynurenine, 3-HAA: 3-hydroxyanthranilic acid, NAD^+^: Nicotinamide adenine dinucleotide (oxidized form), CNS: Central nervous system.

The isoenzymes Indoleamine-2,3-Dioxygenase 1 (IDO1) and Tryptophan 2,3-Dioxygenase (TDO) catalyze the first and rate limiting step of the essential amino acid TRP to Kynurenine (KYN) [13]. While TDO is mainly expressed in the liver, IDO1 can be expressed in various tissues upon stimulation with inflammatory cytokines, such as Interferon-γ and Interleukin-6 [15]. The result is an increase of KYN, which is further metabolized to Kynurenic acid (KYNA) or Quinolinic acid (QUINA) [13]. The latter stimulates N-methyl-D-aspartate (NMDA) receptors in the central nervous system with the potential to induce neuronal excitotoxicity, which is one of the key pathological mechanisms of AD [16]. A simplified visualization of the KP is presented in Fig. 1.

The central dysregulation of the KP also seems to manifest in the blood stream, since accumulated serum QUINA levels [17] and serum Kynurenine to Tryptophan (KYN/TRP) ratios [14] (a surrogate marker of IDO1 activity) were observed in AD patients and women with high cortical amyloid-β burden [11, 18]. In contrast, KYNA rather has a neuroprotective effect in the central nervous system by inhibiting NMDA neurotransmission [16]. Interestingly, reduced plasma KYNA levels were found in AD patients [19].

The dysregulation of the KP and its neurotoxic/neuroprotective downstream metabolites might also affect cognitive functions. In a large cross-sectional study, increased serum KYN/TRP ratios were associated with poor memory performance [20] in elderly people. In another study, higher serum QUINA levels were correlated with worse executive functions in elderly at risk of dementia [21].

Regular exercise has been found to reduce the risk of AD, maintain cognitive performance, and positively influence the KP in healthy populations [22–24]. Metabolically demanding exercise (such as aerobic and strength training) was investigated most frequently in this field. It has the potential to reduce systemic inflammation [25], which results in decreased activity of enzymes regulating the KP [26].

Interestingly, evidence from animal studies suggest a superior effect of exercise and sensory enrichment (known as enriched environment) on neuroplasticity compared to exercise alone [27, 28]. The idea is supported by recent studies in humans, which conducted multicomponent [29] or dancing interventions [30, 31]. In accordance, a recent systematic review suggested that the metabolic demands of exercise are not the sole factor to improve cognitive performance in elderly people [32]. Golf might have similarities to an enriched environment as well, because it combines physical, sensory, cognitive, and social components [33, 34]. Golf provides a low to moderate intensity profile, although walking a golf course contains periods with higher and lower intensities [33]. Besides cardiovascular activity, learning the golf swing requires high demands on hand-eye coordination, static postural as well as sensorimotor control [34, 35]. During a game, strategic planning, maintaining and manipulating information, as well as adapting to changing environmental conditions illustrate the cognitive demands of golf, including working memory, attention [34] as well as cognitive flexibility. In the social domain, regular golfing facilitates the establishment of social connections and relationships [33]. In sum, the multidimensional profile of learning to play golf might have the potential to positively affect SMC-related cognitive decline and biological changes in elderly people. However, because of the challenging profile, it remains unclear if elderly people with cognitive problems are able to learn the sports.

Few studies investigated the effects of learning golf on cognitive functions in elderly people. Shimada et al. (2018) [34] reported improvements in immediate and delayed logical memory after a 24-weeks golf intervention with healthy older adults from Japan. Another research group found an increased visual imaginary ability after 20 sessions of golf training in patients with stroke [36].

This study aimed to examine the feasibility of learning to play golf over 22 weeks and its effects on cognitive functions and the regulation of peripheral KP metabolites in elderly people with SMC with no experience in playing golf.

## Materials and methods

The study is reported in accordance with the Consolidated Standards of Reporting Trials (CONSORT) recommendations of 2010 for pilot and feasibility trials [37].

It was designed as a 22-week randomized controlled trial and conducted between May and December 2018 at the Institute of Sports Medicine at Paderborn University. The protocol was approved by the ethics committee of the “Westfälische Wilhelms-Universität Münster”. Written informed consent to participate in the study was obtained by each participant before enrollment and was in accordance with the Declaration of Helsinki. Participants were not provided any payment, but the golf group received complimentary golf training.

The trial was retrospectively registered (02/07/2018) at the German Clinical Trials Register (DRKS00014921).

### Screening and eligibility

Participants were recruited locally via newspapers, social media advertisements and at organizations providing leisure activities for seniors. Exclusion criteria were (1) younger than 60 years, (2) answer “no” to the question: “Do you have subjective memory complaints?” (3) significant experiences in golf (i.e., proficiency certificate or handicap) (4) diagnosed neurological or mental disease or other impairment of physical abilities.

### Procedure and randomization

After inclusion, participants were individually scheduled for baseline data collection before randomization on two different days. At day one, the acquisition of medical history and sociodemographic data as well as the neuropsychological testing took place. On the second day, participants underwent a blood sample collection and an endurance performance test. To account for differences in physical activity habits between groups’ participants were asked to fill in a questionnaire (Physical Activity Scale for Elderly, PASE, [59]) for two different weeks before randomization and during the 22-week intervention.

After baseline assessments, participants were matched according to age, gender, PASE-score, and Alzheimer’s Disease Assessment Scale - Cognitive Subscale (ADAS-Cog) score [38]. For the matching procedure, the method of randomly permuted blocks was applied to allocate participants with similar characteristics to the golf or control group (1:1 ratio). If a block consisted of one participant or if the number of participants in one block was uneven, the participant was allocated to the golf group. A researcher who was not involved in the neuropsychological tests, blood collection, or golf intervention (FvdB) carried out the random sequence generation and group allocation.

**Fig. 2:**
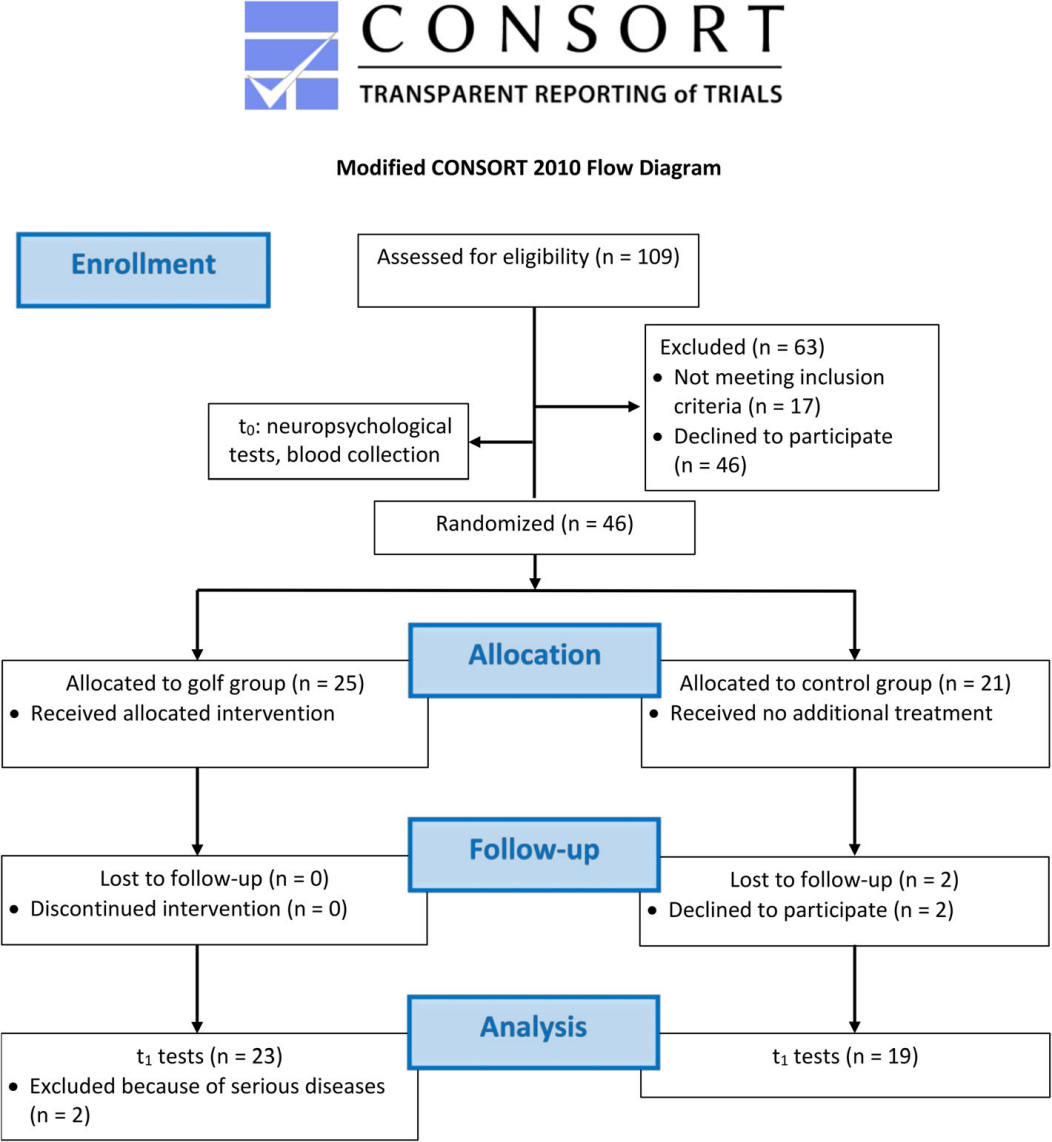
Modified CONSORT flow chart of the study.

A CONSORT flow chart of the study is presented in Fig. 2.

### Subjective memory complaints

According to current guidelines [39] a self-designed questionnaire without further formal cognitive testing was used to characterize people with SMC. It included the items memory complaints in daily life (yes/no), serious worries about memory complaints (yes/no) and the onset of the complaints (years). The presence of depressive symptoms and anxiety was evaluated with the Beck’s Depression Inventory (BDI) [40].

### Golf intervention

The golf training was performed at Paderborn University Golf Club. The golf training sessions were planned, supervised, and mostly conducted by a fully qualified professional golf trainer of the Professional Golfer Association (PGA). In addition, three other golf trainers aided and provided the training sessions. A maximum of 13 participants who were instructed by two trainers was allowed per session. The golf training consisted of three sessions per week, each lasting 60 min over a period of 22 weeks. Two of three sessions were supervised and instructed by trainers. The third session was not supervised, but participants were asked to practice the previously acquired skills independently at the driving range.

The supervised golf program included 18 practice sessions and 25 sessions at the driving range. All sessions started with a short warm-up (10 min), which consisted of coordination and stretching exercises. In the practice sessions (week 1 to 8), participants learned basic golf techniques, starting with putting and chipping. After 5 weeks, pitching was introduced and practiced and after 7 weeks, participants learned the full golf swing. Golf trainers gave individual feedback to improve the techniques, e.g. via videos or verbal instructions during the sessions. At week 9, participants started to practice at the driving range. All trainers encouraged the participants to practice the golf techniques at home in front of a mirror, to learn the golf rules and to interact with the other participants.

### Control group

The control group was asked to keep their lifestyle and sports activities unchanged for the course of the study. Each control subject was asked to fill out PASE questionnaires every week to monitor any changes.

### Primary outcomes: Feasibility and general cognitive performance

To elucidate if learning to play golf is feasible for elderly people with cognitive problems, the golf group underwent a graded technical exam only at the end of the intervention. This exam is equivalent to the German license to play golf. It included the assessment of the golf techniques putting, chipping, pitching, and the full golf swing performed by the professional golf trainer. A score of 20 points could be achieved for each technique, resulting in a total score of 80 points. Therefore, a higher score was related to better golf performance. Attendance rate was measured by the average number of sessions which were conducted by all participants of the golf group compared to the absolute number of conducted trainings. Safety was measured by adverse events and drop-outs related to the golf intervention.

The neuropsychological tests were conducted at baseline (t_0_) and after the 22-week intervention (t_1_) by trained study staff not involved in the intervention and blinded to the group assignment. Blinding was ensured by asking participants not to disclose their group assignment to the outcome assessors. General cognitive functions were assessed with the ADAS-Cog [38], which was sensitive to the effects of physical activity in elderly people with SMC in another study [41].

### Secondary outcomes: Specific cognitive functions, physical evaluation and the KP

In addition, three different tests of specific cognitive functions were conducted and consisted of visual-spatial working memory (assessed with Corsi Block Tapping Task), and executive functions (assessed with INHIB Response Inhibition and the Trail Making Test part B, respectively). Except for the ADAS-Cog, all tests were part of the automated computer-based Vienna Test System Version 6.82.000 (Schuhfried GmbH, Mödling, Austria).

To assess endurance performance, the 6-Minute-Walk-Test (6MWT) was conducted [42].

Blood samples via venipuncture were obtained during early and late morning (8–12 am) to investigate the KP and IL-6. Participants were asked to avoid physically demanding activities on the same morning before the blood sample collection. The serum samples were left to clot at room temperature for 30 min. After that, samples were centrifuged for 10 min at 1800 g and frozen at -31 °C until transportation (max. 2 weeks later).

Blood samples were processed and analyzed as described in detail in Joisten et al. 2020 [43]. High performance liquid chromatography (HPLC) and tandem mass spectrometry (MS/MS) were used to analyze serum concentrations of KP metabolites (KYN, TRP, QUINA, KYNA). Ratios of KYN to TRP, QUINA to KYNA, QUINA to TRP and QUINA to KYN were calculated to indicate changes in the degradation steps of the KP.

IL-6 was measured using the Quantikine high sensitive IL-6 Enzyme-linked Immunosorbent Assay (ELISA, R&D Systems, Minneapolis, USA) according to the manufacturers protocol.

### Statistical analysis

The sample size calculation was conducted with G*Power [44] and was based on the randomized controlled trial of Lautenschlager et al. (2008) [41]. The standardized mean difference for ADAS-Cog was − 1.22. The drop-out rate was set to 20 %. We estimated that a sample size of 46 participants (23 in each group) would provide 95 % power for detecting a significant group difference.

All statistical analyses were conducted per protocol and with the statistical software SPSS version 23 for Windows (IBM, Armonk, NY, United States). Baseline differences between groups regarding anthropometric data, the prevalence of risk factors and physical performance were checked with either independent t-test, Mann-Whitney-U-test or χ^2^-test for categorical variables. Data were z-transformed and statistical outliers (defined as ± 3 SD from the mean) were excluded for each variable. Normal distribution was checked with the Shapiro-Wilk test (p > 0.05), and homoscedasticity with Levene’s test (p > 0.05). Baseline-adjusted Analyses of Covariance (ANCOVAs) were conducted to determine significant group differences for cognitive and blood biomarkers, with group (golf, control) and time (baseline, post) as the main factors. The specific baseline values as well as age were used as covariates for all analyses. Additionally, the change score of BMI (t_1_-t_0_) was used as a covariate for the analysis of the KP, since visceral fat mass is known to induce systemic low-grade inflammation [23]. Significant time*group interactions were post-hoc analyzed with pairwise Bonferroni-adjusted tests. Due to the explorative character and the small sample size, a larger p-value spectrum was considered for the KP data only [45]. Of note, not all parameters fulfilled the criteria for parametric tests (not normal distributed, heteroscedasticity). ANOVA was shown to be robust against violation of normal distribution, especially when group sizes were over 10 [46]. Transformation of variables to achieve assumptions for parametric tests was also performed, but not associated with different results.

An effect size between Cohen’s d ≤ 0.2 was considered a small effect, Cohen’s d 0.2 to 0.5 a medium effect and Cohen’s d > 0.8 a large effect [47]. To explore the association between compliance (overall number of sessions, number of attended third unsupervised training sessions only) and biological markers (IL-6, KP metabolites), Spearman-rank correlations were calculated, with a significance level of p < 0.05. The obtained p-values from the correlation analysis were corrected with the False Discovery Rate (FDR) procedure [48]. Due to the explorative character of the study, corrected and uncorrected p-values of the correlation analysis were reported.

## Results

### Baseline characteristics

No statistically significant baseline differences between groups regarding age, gender, education, BDI-score, ADAS-Cog-Score, as well as cardiovascular risk factors and physical outcomes were detected. According to the BDI [40], two participants reported symptoms of mild depression at baseline (16 points), but were not diagnosed with depression or bipolar disorder. The other participants had no clinically relevant symptoms of depression (< 12 points). An overview of baseline characteristics is presented in Table 1.

**Table 1:** Demographic information at baseline of analyzed participants (*n* = 42)

	Golf group (*n* = 23)	Control group (*n* = 19)	*p*-value	Cohen’s d
Age (years)	67.87 ± 4.7	67.89 ± 3.9	0.95	0.05
Gender				
male	10 (43.5 %)	9 (47.4 %)	0.51	
female	13 (56.5 %)	10 (52.6 %)		
Handedness				
right	22 (95.7 %)	19 (100 %)	0.53	
left	1 (4.3 %)	0		
Formal education (years)	14.4 ± 4.5	12.26 ± 3.6	0.13	0.59
BDI (score)	4.7 ± 4.4	4.4 ± 3	0.93	0.10
ADAS-Cog (score)	8 ± 3.55	7.68 ± 3.41	0.71	0.09
**Cardiovascular risk factors**				
BMI	26.3 ± 3.4	26.2 ± 3.9	0.44	0.02
Current smoker	3 (13 %)	2 (10.5 %)	0.55	
Heavy smoker	0 (0 %)	1 (5.3 %)	0.48	
Diabetes mellitus type 1	2 (8.7 %)	1 (5.3 %)	0.54	
Diabetes mellitus type 2	1 (4.3 %)	1 (5.3 %)	0.73	
Hypertension	12 (52.2 %)	7 (36.8 %)	0.26	
Heart disease	4 (17.4 %)	3 (15.8 %)	0.55	
**Physical outcomes**				
6-min-Walk-Test (m)	651.37 ± 121.32	627.73 ± 99.67	0.48	0.24
PASE (score)	181.11 ± 59.52	153.37 ± 57.05	0.15	0.48

Quantitative variables were expressed as means ± standard deviations and categorical variables were expressed as numbers and percentage values

Group differences were tested with Mann-Whitney-U for ordinal scaled variables, with independent t-test for metric variables, and with χ^2^ tests for categorical variables. *BDI *Becks-Depression-Inventory; *ADAS-Cog* Alzheimer’s Disease Assessment Scale - Cognitive Subscale, *BMI *Body Mass Index; *PASE *Physical Activity Scale for Elderly, *p* ≤ 0.05 = statistical significance

### Subjective memory complaints

All participants of both groups reported SMC in daily life. 26 % (golf, *n* = 6) and 32 % (control, *n* = 6) reported serious worries about the complaints. The onset of memory complaints was 2.6 years ago in the golf group (± 2 years) and also in the control group (± 2.6 years). After the intervention, 35 % (*n* = 8) of the golf group reported an improvement of these symptoms, while no improvement was reported in the control group. 5.3 % (*n* = 1) of the control group and no one in the golf group reported a worsening during the 22 weeks. 65 % (*n* = 15) of the golf group and 95 % (n = 18) of the control group reported unchanged symptoms.

### Primary outcomes: Feasibility and general cognitive performance

**Fig. 3:**
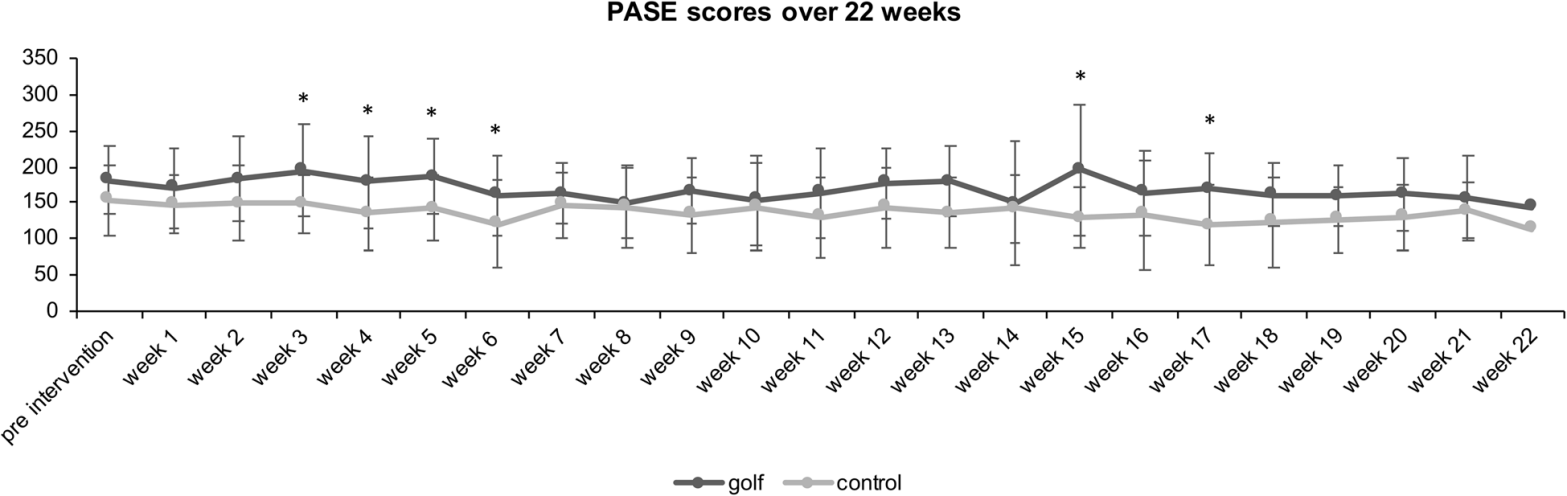
Mean ± Standard Deviation (SD) Physical Activity Scale for Elderly (PASE) scores before the intervention and for every week in each group during the intervention. * indicate significant group differences at *p* < 0.05. 69 % of all PASE questionnaires were filled out in both groups. The lowest number of returned PASE questionnaires was at week 20 (n = 20), the highest number at weeks 1 and 4 (*n* = 35).

A CONSORT Flow Diagram of the study is presented in Fig. 2. All participants (n = 25) of the golf group and 19 participants of the control group took part in the assessments at both time points. Two participants of the control group dropped out without provided reasons. The development of serious diseases during the intervention (brain tumor and motor neuron disease), which were not related to the study, required the exclusion of two participants of the golf group from all analyses. There were no adverse events related to the golf intervention during the study. The overall attendance rate in the golf group was 75 % (48 ± 9.9 of 65 sessions) and 70 % (15 ± 5.2 of 22 sessions) for the third training session only. All subjects who undertook the golf exam in the end passed (20/23) it, but three subjects were unable to attend the test due to limited time. 69 % of all PASE questionnaires were filled out during the observation period in both groups. Self-reported activity levels during the study are presented in Fig. 3.

No significant time or time*group interactions were found for ADAS-Cog (*p* = 0.613, F = 0.260, Cohen’s d = 0.17). An overview of the statistical results as well as means and standard deviations at t_0_ and t_1_ is presented in Table 2.

**Table 2:** Descriptive data indicated as mean and standard deviation (SD) at baseline (t_0_) and after the intervention (t_1_)

outcome	group	n	t_0_ (mean, SD)	t_1_ (mean, SD)	ANCOVA time*group (p, F, Cohen’s d)	post-hoc analysis (p, 95 % CI)
ADAS-Cog^1^ (score)	golf	22	7.45 (2.46)	6.82 (2.67)	0.613, 0.260, 0.17	
	control	19	7.68 (3.40)	6.58 (2.97)		
Corsi forward^1^ (no. of sequences)	golf	22	7.91 (1.31)	7.77 (2.02)	0.641, 0.221, 0.16	
	control	19	6.74 (2.13)	7.16 (2.43)		
Corsi backwards^1^ (no. of sequences)	golf	23	6.48 (2.35)	6.65 (2.77)	0.921, 0.010, 0	
	control	19	6.26 (2.96)	6.42 (3.15)		
TMT B time^1^ (s)	golf	22	49.44 (18.87)	47.58 (12.57)	0.305, 1.085, 0.35	
	control	18	49.81 (22.25)	52.66 (29.03)		
INHIB reaction time^1^ (ms)	golf	22	0.37 (0.05)	0.36 (0.05)	0.975, 0.000, 0	
	control	19	0.35 (0.04)	0.35 (0.05)		
INHIB correct responses^1^ (%)	golf	22	91.36 (6.84)	95.27 (3.24)	0.012*, 7.050, 0.89	0.000*, (2.075, 4.884)
	control	18	92.87 (4.27)	93.07 (4.41)		0.349, (− 0.827, 2.280)
KYN^2^ (µmol/L)	golf	22	1.69 (0.55)	1.84 (0.74)	0.418, 0.671, 0.28	
	control	17	2.04 (0.99)	2.19 (0.85)		
TRP^2^ (µmol/L)	golf	22	127.48 (25.68)	129.67 (16.39)	0.464, 0.548, 0.26	
	control	17	129.67 (20.90)	126.56 (21.13)		
KYN/TRP ratio^2^	golf	22	0.014 (0.005)	0.014 (0.005)	0.087, 3.108, 0.61	
	control	17	0.016 (0.006)	0.017 (0.006)		
KYNA (µmol/L)^2^	golf	22	0.093 (0.023)	0.095 (0.031)	0.926, 0.009, 0	
	control	17	0.105 (0.048)	0.105 (0.039)		
KYNA/KYN ratio^2^	golf	22	0.058 (0.02)	0.057 (0.02)	0.598, 0.284, 0.18	
	control	17	0.057 (0.02)	0.054 (0.03)		
QUINA (µmol/L)^2^	golf	21	0.72 (0.24)	0.67 (0.22)	0.224, 1.537, 0.43	
	control	17	0.71 (0.24)	0.73 (0.19)		
QUINA/KYNA ratio^2^	golf	21	8.20 (2.32)	7.38 (1.69)	0.227, 1.513, 0.43	
	control	17	7.43 (2.65)	7.64 (2.92)		
QUINA/TRP	golf	21	0.0058 (0.002)	0.0051 (0.001)	0.022*, 5.769, 0.84	0.022*, (0, 0.001)
	control	17	0.0057 (0.002)	0.0059 (0.002)		0.527, (-0.001, 0)
QUINA/KYN	golf	21	0.456 (0.145)	0.406 (0.154)	0.869, 0.028, 0.06	
	control	17	0.394 (0.141)	0.364 (0.107		
IL-6 (pg/ml)^2^	golf	22	1.54 (0.59)	1.82 (0.97)	0.607, 0.270, 0.18	
	control	15	2.05 (1.17)	2.16 (1.33)		
6-Min-Walk-Test^1^ (m)	golf	23	651.65 (115.75)	653.58 (153.82)	0.439, 0.613, 0.26	
	control	16	646.21 (91.12)	673.78 (106.33)		
PASE (score)^1^	golf	21	180.86 (59.02)	171.05 (45.72)	0.056, 3.888, 0.66	
	control	19	153.37 (57.05)	137.50 (37.91)		

Results of baseline-adjusted ANCOVA are presented (time, time*group) with Bonferroni-corrected pairwise post-hoc analysis for each parameter. * indicate statistical significant changes or differences at *p* < 0.05. *ADAS-Cog* Alzheimer’s Disease Assessment Scale - Cognitive Subscale, *TMT* Trail Making Test, *INHIB* Response Inhibition, *KYN* Kynurenine, *TRP* Tryptophan, *KYNA* Kynurenic acid, *QUINA* Quinolinic acid, *IL-6* Interleukin 6, *PASE* Physical Activity Scale for Elderly, *FDR *False Discovery Rate (Benjamini Hochberg Procedure) ^1^adjusted for age, ^2^ adjusted for age and mean change of BMI (t_1_-t_0_)

### Secondary outcomes: Specific cognitive functions, KP metabolites, and physical performance

No significant time or time*group interactions were found for TMT B or INHIB reaction time. Significant time effects were found for Corsi forward (*p* = 0.033, F = 4.888, Cohen’s d = 0.16) and Corsi backwards (*p* = 0.023, F = 5.620, Cohen’s d = 0). However, after Bonferroni-adjusted pairwise post-hoc analysis no significant changes in both groups for these parameters remained. A significant time*group interaction was found for INHIB correct responses (*p* = 0.012, F = 7.050, Cohen’s d = 0.89), while reaction time remained stable in both groups. Bonferroni post-hoc analysis revealed a significant increase in INHIB correct responses in the golf group compared to the control group (*p* = 0.012, 95 % CI 0.653, 4.834).

No significant time or time*group interactions were found for KYN, TRP, KYNA, QUINA, KYNA/KYN, QUINA/KYNA or QUINA/KYN ratio, IL-6 and for the 6-Min-Walk-Test, but for QUINA/TRP (*p* = 0.022, F = 5.769, Cohen’s d = 0.84) in favor of the golf group (*p* = 0.022, 95 % CI 0, 0.001).

**Fig. 4:**
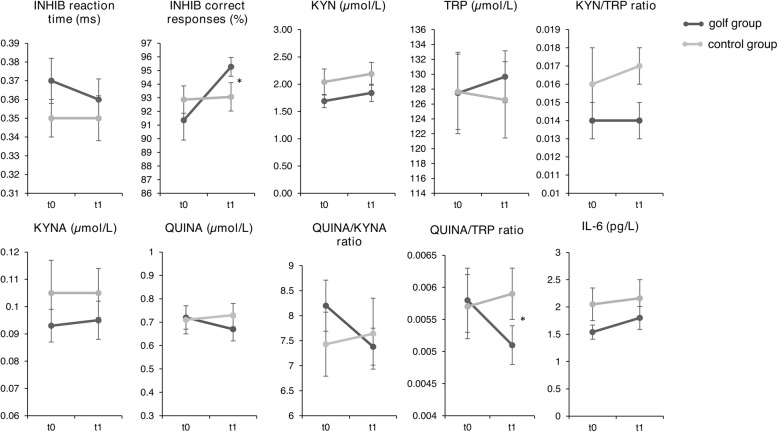
Raw data (means) with standard error (SE) in both groups at t_0_ and t_1_. * indicate statistical significant time*group interactions at *p* < 0.05. KYN: Kynurenine, TRP: Tryptophan, QUINA: Quinolinic acid, KYNA: Kynurenic acid, INHIB: Response Inhibition task, BDNF: Brain-derived neurotrophic factor, IL-6: Interleukin-6.

A trend towards a significant time*group interaction was found for KYN/TRP ratios (*p* = 0.087, F = 3.108, Cohen’s d = 0.61). Another almost significant group*time interaction was found for PASE (*p* = 0.056, F = 3.888, Cohen’s d = 0.66). Significant interaction effects as well as trends within the KP are visualized in Fig. 4.

**Fig. 5:**
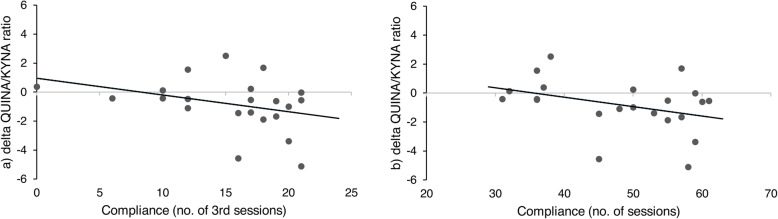
Results of Spearman correlation analyses. **a** + **b**: Association of compliance (number of attended sessions) with delta Quinolinic acid to kynurenic acid ratios (QUINA/KYNA ratios, t1 - t0). **a**: only number of third sessions attended) and delta QUINA/KYNA ratios, **b** number of all attended sessions and delta QUINA/KYNA ratios. **a** r_s_ = − 0.443, *p* = 0.039, **b** r_s_= -0.400, *p* = 0.065. Of note, the correlation between compliance (no. of 3rd sessions) and delta QUINA/KYNA ratios was not significant after correcting for type 1 errors (*p* = 0.039 > 0.005

Exploratory Spearman correlation analysis showed a significant negative correlation of delta QUINA/KYNA ratio (t_1_-t_0_) with adherence to the third training session in the golf group (*p* = 0.039, r_s_ = -0.443). Of note, the correlation was not significant after FDR correction. An association between the achieved points of the golf exam and the KP was not found. Results of the correlation analyses are presented in Fig. 5.

## Discussion

The aim of this study was to elucidate the feasibility of a 22-weeks golf intervention on the driving range and its effects on cognitive functions and the KP in elderly people with SMC without prior golf experience. We could show that elderly people with cognitive problems were able to learn golf, although it is a complex and difficult sport to learn. No adverse events related to the golf intervention were reported, and no participant in the golf group dropped out. Overall adherence to the golf training was 75 %, which can be considered as acceptable. Two participants of the control group dropped out, as they made their participation contingent on allocation into the golf group. Of note, this might have influenced comparability of both groups, e.g. regarding physical activity levels (PASE). Three participants were not able to attend the golf exam due to organizational reasons. All other participants passed it (20/23). Therefore, for people with cognitive problems, findings support the idea that learning to play golf is feasible and safe.

Results of this pilot study revealed no significant effects of learning to play golf on general cognitive performance, working memory or other measures of executive functions compared to the control group. Only the number of correct responses on the INHIB task increased significantly in the golf group compared to the control group, while the reaction time remained unchanged. For the INHIB task, participants should only react when a certain stimulus is presented (Go/NoGo). Golfers therefore made less omission (missing reaction) and commission errors (wrong reaction) compared to the control group throughout the task. These findings suggest a small effect on sustained attention.

Shimada et al. (2018) [34] found an improvement in logical memory after 24 weeks golf intervention, which was not an outcome in this study. Learning to play golf requires high demands on motor and cognitive abilities [35] and less on cardiovascular abilities (compared to endurance training). In addition, the golf training consisted mostly of whole-body-coordination exercises at the driving range, which is also indicated by an unchanged endurance performance in the golf group. The findings are in accordance with Voelcker-Rehage et al. (2011) [49], who found a higher accuracy during a Flanker and a Visual Search task in a coordination exercise group. Thus, our results might not reflect the multidimensionality of playing golf, which also includes cardiovascular demands that is induced by walking over the fairway [33]. Evidence from studies investigating the effects of dancing (which is possibly comparable to golf because of its multidimensionality) revealed increases in attention only at 6 months of dance training, whereas episodic memory performance increased after 18 months of training [30]. Improvements in cognitive functions were reportedly not linear [30], and the positive effects of a multidimensional activity on cognitive functions might not be present at 22 weeks of practice. In this study, only data from the 22-week observation is available, which impedes the long-term investigation of all demands of golf on cognitive functions.

On a biological level, learning to play golf showed a positive, but non-significant influence on peripheral KP regulation, which could be of interest for future studies. A significant group*time interaction was only found for QUINA/TRP ratios in favor of the golf group. Descriptive data indicated that KYN levels as well as KYN/TRP ratios remained stable during the intervention in the golf group, but increased almost significant in the control group, suggesting enhanced IDO1 activity. Of note, KYN/TRP ratios could also increase by cortisol due to higher stress levels in the control group, which in turn influences the expression of TDO [23, 50]. Interestingly, the KYN/TRP ratios observed in this study are lower compared to findings from cross-sectional studies, e.g. in elderly people (median KYN/TRP ratio 0.025, interquartile range 0.006, [20]) and in AD patients (median KYN/TRP ratio 0.034, interquartile range 0.008, [17]). Besides substantially increased KYN/TRP ratios that have been described in neurodegenerative diseases [51], the participants in Solvang et al. [20] were on average 10 years older compared to this sample, which might be one explanation for the higher values. To the best of our knowledge, no cut-off value exists yet that indicates an increased risk for future cognitive decline or AD.

The non-significant increase of KYN/TRP ratios could also be the result of decreased habitual physical activity in the control group during the observation period, as indicated by PASE scores (see Fig. 2). The activity associated with regular golf training therefore might have the potential to counteract age or AD-related changes of IDO1 activity. This is supported by the non-significant decrease of serum QUINA levels as well as serum QUINA/KYNA ratios golf group only, which might indicate a reduction of Kynurenine 3-monooxygenase (KMO) activity [50, 52]. IDO1 and KMO activity are both driven by inflammatory states [13, 53]. Of note, the findings did not reach statistical significance, and should therefore be replicated in a larger sample. In addition, it cannot be ruled out that the results are affected by other enzymes of the KP, such as Kynureninase or Kynurenine aminotransferase (KAT).

We also found a negative association between delta QUINA/KYNA ratios and the number of golf training sessions, indicating a positive influence of training frequency on the KP. Of note, the correlation was not significant after Benjamini Hochberg-correction of type 1 error and should also be interpreted carefully.

One randomized controlled trial exists which investigated the effects of a multicomponent exercise intervention (endurance, coordination, balance, flexibility, strength) in elderly at risk of dementia. Küster et al. [21] did not find effects of a 10-week multicomponent exercise intervention on KP metabolites either. Similar to our intervention, the metabolic demands might have been too low to trigger mechanisms that were described regarding the positive effects of exercise on the KP [23], as KYN remained unchanged in the golf group. The positive effects of learning to play golf on the KP might be rather mediated by reduced systemic inflammation, stress or other mechanisms, which are reported effects of physical activity [25, 54].

Our study has some limitations, which should be considered when interpreting the results. First, the sensitivity of the ADAS-Cog might have been too low to detect cognitive changes in this cohort. However, this test was chosen as the primary outcome to allow comparability to previous studies with similar cohorts (e.g. [55–57]). Second, the sample was active (according to the PASE questionnaire, [58, 59]), physically fit (according to the 6MWT, [42]) and well educated (13.5 ± 4.2 years on average), which indicates a selection bias that may affect the generalizability of the results. Of note, it would be of great interest to motivate sedentary elderly people, which are not already physically active, to take part in such a program. In recent years, accessibility of golf was increased by modifying rules of the game, the golf course as well as the equipment for people with various impairments [60]. Moreover, playing golf can facilitate establishment of social relationships, which in turn increases motivation to continue with the sports.

Third, self-reported and cognitive outcomes in this study might be prone to reflect placebo effects or other factors related to the intervention, such as an increased social interaction, because blinding of the participants was not possible and there was no adequate treatment in the control group. The results should therefore be interpreted carefully.

Accordingly, we did not achieve the calculated sample size because many of the screened subjects made their participation in the study contingent on a randomization into the golf group and therefore had to be excluded. The study is therefore underpowered, which might have led to the absence of the effects of golf on neuropsychological outcomes and the KP. Thus, our findings should be replicated in a larger, more representative sample and rather be interpreted as preliminary results. Subgroup analyses of the effects of golf on cognitive functions and the KP accounting for particular concerns and gender differences would have also been useful [4]. In this context, it was recently shown that elderly women with high cortical amyloid-β burden also had higher serum KYN levels and KYN/TRP ratios [11]. Serious concerns about SMC were further associated with quantitative amyloid-β deposition [10] and it was suggested, that the decline in estrogen levels after menopause might increase the vulnerability to KP activity and AD [11]. In our study IL-6 was used as a surrogate marker for inflammatory processes that may influence KP regulation via upregulation of IDO or TDO. However, further studies should include a broader variability of markers of neurodegeneration, neuroinflammation and neuroplasticity.

People with SMC were diagnosed according to guidelines published in [39]. There is some evidence that self-reported SMC can predict future cognitive decline [61], but findings are controversial [62]. Thus, it remains unclear if the study population is relevant for AD or if SMC had other etiologies.

Participants were not scheduled at the exact same time of the day at baseline and after the intervention for the blood sample collection and chronobiological effects might not be fully excluded. However, moderate to strong correlations of t_0_ and t_1_ IL-6 and KP parameters do not support relevant effects. Participants were not fasted during blood sample collection, which might have influenced the outcome measures as well. Therefore, future studies should conduct measurements with standardized conditions.

In general, causality of central changes of the KP by the golf intervention cannot be assumed, but has to be elucidated in further studies [21].

Since the aim of this study was to assess effects of a complex multicomponent activity in analogy to the enriched environment animal model, we are limited in concluding which stimulation (cognitive, social, sensory, motor) had the largest effects on cognitive outcomes and the KP or whether the combination of all the above is most effective. However, the current literature supports positive effects of recreational and physical activities in natural green environments on mental health in elderly people [63] and attention performance [64].

## Conclusions

The findings of this randomized trial support the idea that learning the challenging sports golf with a multidimensional profile is feasible and safe for people with SMC. Preliminary results revealed improved attention performance and a trend towards a positive influence on the regulation of the KP in the golf group. To elucidate the effects of long-term golf training and the potential to reduce cognitive decline and associated changes in the KP, studies should replicate the findings in a larger and less active sample of elderly people with SMC over a longer period, including higher metabolic demands by playing on the golf course.

## Data Availability

The datasets used and/or analysed during the current study are available from the corresponding author on reasonable request.

## Abbreviations

AD: Alzheimer’s Disease
ADAS-Cog: Alzheimer’s Disease Assessment Scale – Cognitive Subscale
ANCOVA: Analysis of Covariance
ANOVA: Analysis of Variance
BDI: Beck’s Depression Inventory
BMI: Body-Mass-Index
CNS: Central nervous system
CONSORT: Consolidated Standards of Reporting Trials
ELISA: Enzyme-linked Immunosorbent Assay
FDR: False Discovery Rate
HPLC: High performance liquid chromatography
IDO1: Indoleamine-2,3-Dioxygenase 1
IL-6: Interleukin-6
INHIB: Response Inhibition Task
KAT: Kynurenine Amino Transferase
KMO: Kynurenine 3-monooxygenase
KP: Kynurenine Pathway
KYN: Kynurenine
KYNA: Kynurenic Acid
NAD+: Nicotinamide adenine dinucleotide (oxidized form)
PASE: Physical Activity Scale for Elderly
PGC-1α: Peroxisome proliferator-activated receptor gamma coactivator 1-alpha
PGA: Professional Golfers Association
QUINA: Quinolinic Acid
SD: Standard Deviation
SMC: Subjective Memory Complaints
TDO: Tryptophan 2,3-Dioxygenase
TMT: Trail Making Test
TRP: Tryptophan
6MWT: 6-Minute Walk Test
3-HK: 3-hydroxykynurenine
3-HAA: 3-hydroxyanthranilic acid
